# Macrophage heterogeneity in human secondary lymphoid organs

**DOI:** 10.1042/CS20250086

**Published:** 2026-08-13

**Authors:** Elodie Segura

**Affiliations:** INSERM, Université Paris Cité, CNRS, Institut Necker Enfants Malades - INEM, 156 rue de Vaugirard, 75015 Paris, France

**Keywords:** human, lymphoid organ, macrophage

## Abstract

Macrophages play a major role in immune responses and in the maintenance of tissue homeostasis. Most of our knowledge of macrophage biology comes from mouse studies, which have revealed the existence of distinct subpopulations differing in origin, life cycle and tissue-specific features, as well as the influence of tissue perturbations on macrophage heterogeneity. Here, we review the evidence supporting the conservation of these principles in human macrophages, along with the methodological approaches employed, including omics analyses of macrophages from clinical samples, human transplantation studies and studies of monogenic mutations. We also focus on the macrophage populations that are present in human secondary lymphoid organs—namely the spleen, lymph nodes, and tonsils. While some organ-specific features are shared with their mouse counterparts, we highlight important differences between mouse and human lymphoid tissue macrophages in their functional specialization.

## Introduction

Macrophages are essential players in immune responses through pathogen recognition, dead cell clearance and resolution of inflammation, and also play major homeostatic roles including in the control of metabolism and the cross-talk with stromal cells [1,2]. Calculations based on human histology literature, multiplexed imaging studies, and deconvolution of human epigenetic atlases have estimated that macrophages represent minor fractions of immune cells in most human tissues, except for lung and liver [3]. However, macrophages are distributed widely across the body without a dominant presence in any system, and represent around 10% of total immune cells in the human body [3].

Macrophages can be schematically divided into tissue-resident macrophages (TRM) that are usually long-lived and populate steady-state tissues, and short-lived macrophages that are induced upon inflammation and derived from monocytes. Mouse studies employing genetic models have shown that numerous factors impact macrophage diversity and population heterogeneity. Despite methodological limitations, studies of human macrophages from clinical samples indicate that the main principles of macrophage biology are conserved between mouse and human. There are also notable differences, in particular, in secondary lymphoid organs.

## Macrophage heterogeneity—insights from comparative transcriptomics

Mouse studies based on fate-mapping, i.e. cell lineages tracing *in vivo* using genetic marks or fluorochromes, have shown that TRM can originate either from embryonic precursors or from adult monocytes [4,5]. In steady-state mice, TRM are mainly derived from embryonic or perinatal precursors and can self-maintain by proliferating *in situ* [4,5]. Upon aging, long-lived TRM are gradually replenished by incoming blood monocytes [5,6]. In addition, a population of short-lived TRM derived from monocytes is also present in steady-state mucosal tissues, such as intestine and skin, or in serous cavities [5,7–9]. These short-lived TRM are constantly replaced by homeostatic input from circulating monocytes. Based on transcriptomic analyses, phenotypic markers have been proposed to distinguish macrophage populations from different origins [6,10,11]. *TIMD4, LYVE1* and *FOLR2* are proposed markers for embryo-derived long-lived TRM, high expression of MHC class II molecules for long-lived TRM that derive from adult monocytes replenishing the initial population, and *CCR2* for monocyte-derived short-lived TRM. These markers may not apply to all tissues.

Seminal studies in mice comparing the transcriptomic and epigenetic profiles of macrophages from diverse tissues have evidenced the great diversity of TRM, with a core macrophage gene program coexisting with tissue-specific transcriptomic profiles [12–14]. This has led to the notion that TRM identity and function are driven by specific transcription factors whose expression is imprinted by signals from their anatomical niche. In addition, long-lived monocyte-derived TRM were found to gradually adopt the tissue-specific gene profile of the macrophages they replace, through their instruction by cues from the anatomical niche [4,6].

Single-cell RNA-sequencing (scRNA-seq) profiling of macrophage populations can be used to infer their origin based on the similarity of their transcriptional signatures to those of mouse macrophages, and *in silico* predictions of differentiation trajectories. In particular, distinct populations of monocyte-derived and embryo-derived TRM have been identified with this methodology in human non-diseased liver, lung, heart, and kidney [6], colon [15] and uninflamed tonsils [16]. However, a caveat is that transcriptomic profiles of monocyte-derived and embryo-derived macrophages may converge over time when exposed to the same instructing signals from their anatomical niche.

## Macrophage heterogeneity—evidence from transplantation studies and human genetics

The study of organ transplantation is a valuable source of information in humans. Analyses of mismatched transplantation settings have evidenced the existence of macrophage subsets with distinct lifespans. Such studies are possible when donor and recipient differ, for instance, in human leukocyte antigen subtypes or in the expression of the Y chromosome, allowing for the tracking in the transplanted tissue of donor-derived and recipient-derived macrophages over time. Donor-derived macrophages were found to persist from months to years in lung [17,18], skin [19,20], and liver transplants [21]. These long-lived macrophages coexisted with recipient-derived macrophages, presumably derived from incoming recipient monocytes replenishing the tissue. In the heart and intestine, several populations of macrophages with distinct replacement rates were also evidenced, corresponding to CCR2+ short-lived monocyte-derived TRM and CCR2− long-lived TRM [22,23].

Moreover, inborn monogenic mutations also provide insight into the biology of human immune cells. Reports of patients with congenic monocytopenia, due to mutations in GATA2 [24,25] or IRF8 [26], show dramatically reduced levels of circulating monocytes but normal abundance of macrophages in various tissues including skin and broncho-alveolar fluid. This is consistent with early seeding of TRM and maintenance through self-renewal.

## Human tissue macrophages and the adaptation to their niche of residence

Several studies have characterized human macrophages in individual tissues using explants. Transcriptomic profiling and *in situ* imaging have revealed the presence of distinct macrophage populations in sub-tissular anatomical niches in uninflamed liver [27], tonsils [16], and colon [15]. Other studies have analyzed macrophages across multiple sites from the same organ donors by profiling RNA and surface protein expression or the chromatin landscape [28,29] and evidenced the existence of a core macrophage program along with tissue-specific gene expression also in humans. For instance, *MYB*, *CSF1R,* and *MAFB* were found to be expressed by all macrophages across tissues. The importance of niche-derived cues in shaping macrophage identity is further shown by the fact that gene expression is significantly affected upon transfer of human brain macrophages (termed microglia) into an *in vitro* context [30]. In addition, *in vitro* systems of human macrophage differentiation from induced pluripotent stem cells have shown that culture with tissue stromal cells or organoids promotes the expression of tissue-specific genes of the brain [31,32] or colon [33].

Collectively, this body of work indicates that human macrophages comprise different subpopulations with distinct origins, life cycles, and niche-specific features.

## Influence of inflammation on macrophage diversity

Numerous studies in mice have shown that monocytes are massively recruited to tissues upon inflammation and that they differentiate *in situ* into macrophages or dendritic cells [34,35]. In humans, experimental models of acute inflammation, including skin blister [36], experimentally induced allergic rhinitis [37], and inhalation of lipopolysaccharide [38], have also evidenced the rapid recruitment of monocytes to inflamed tissues. Increased abundance of monocytes was also observed in tonsils from patients with severe acute respiratory syndrome coronavirus 2 infection [39]. Moreover, transcriptomic profiling of healthy versus diseased tissues has documented the enrichment of macrophages with monocyte-related gene signatures in inflamed gut [40,41], in liver cirrhosis [42], in the lungs of COVID-19 patients [43,44], in the heart of patients with cardiomyopathies [45], and in the synovial tissue of rheumatoid arthritis patients [46]. These observations suggest that, similar to the mouse, monocytes can differentiate *in situ* in inflamed tissues in humans. The molecular mechanisms underlying the commitment toward macrophage versus dendritic cell fate and the molecular regulators of monocyte differentiation are reviewed elsewhere [34,47].

Exposure to microbial products can also induce a phenomenon of innate memory in macrophages, whereby subsequent responses to infection and inflammation are enhanced [48,49]. Mouse and human studies have shown that transient inflammatory cues can be converted in precursor cells in the bone marrow into persistent changes at the epigenetic level, a phenomenon termed ‘central trained immunity’ [50,51]. A similar process has been evidenced at the local level in inflamed tissues. While the majority of monocyte-derived macrophages that develop during inflammation are short-lived and disappear within a few days after resolution of inflammation, some of the inflammation-experienced monocyte-derived macrophages remain in the tissue for a prolonged period of time (up to several months) and retain a more inflammatory transcriptomic profile driven by persistent epigenetic modifications, representing ‘peripheral trained immunity’ [52–59]. Of note, this aspect may be underestimated in mouse studies. Indeed, laboratory mice are kept in super-hygienic conditions, and their immune system has been shown to be more similar to human newborns than adults [60]. In particular, the spleen of wild mice harbors a significantly larger proportion of activated monocytes and macrophages compared with laboratory mice, which could be due to more frequent pathogen exposure [61].

In humans, the persistence of inflammatory features in inflammation-experienced macrophages was observed in cardiac macrophages from heart failure patients [23]. In addition, patients who have recovered from severe sepsis were found to have a lower incidence of cancer than matched non-severe infection survivors, which was correlated with persistent changes in chemokine expression in alveolar macrophages and associated increased recruitment of anti-tumoral T cells [62]. These observations suggest that human macrophages can be imprinted by inflammatory signals they receive in the tissue.

## Spleen macrophages

**Figure 1:**
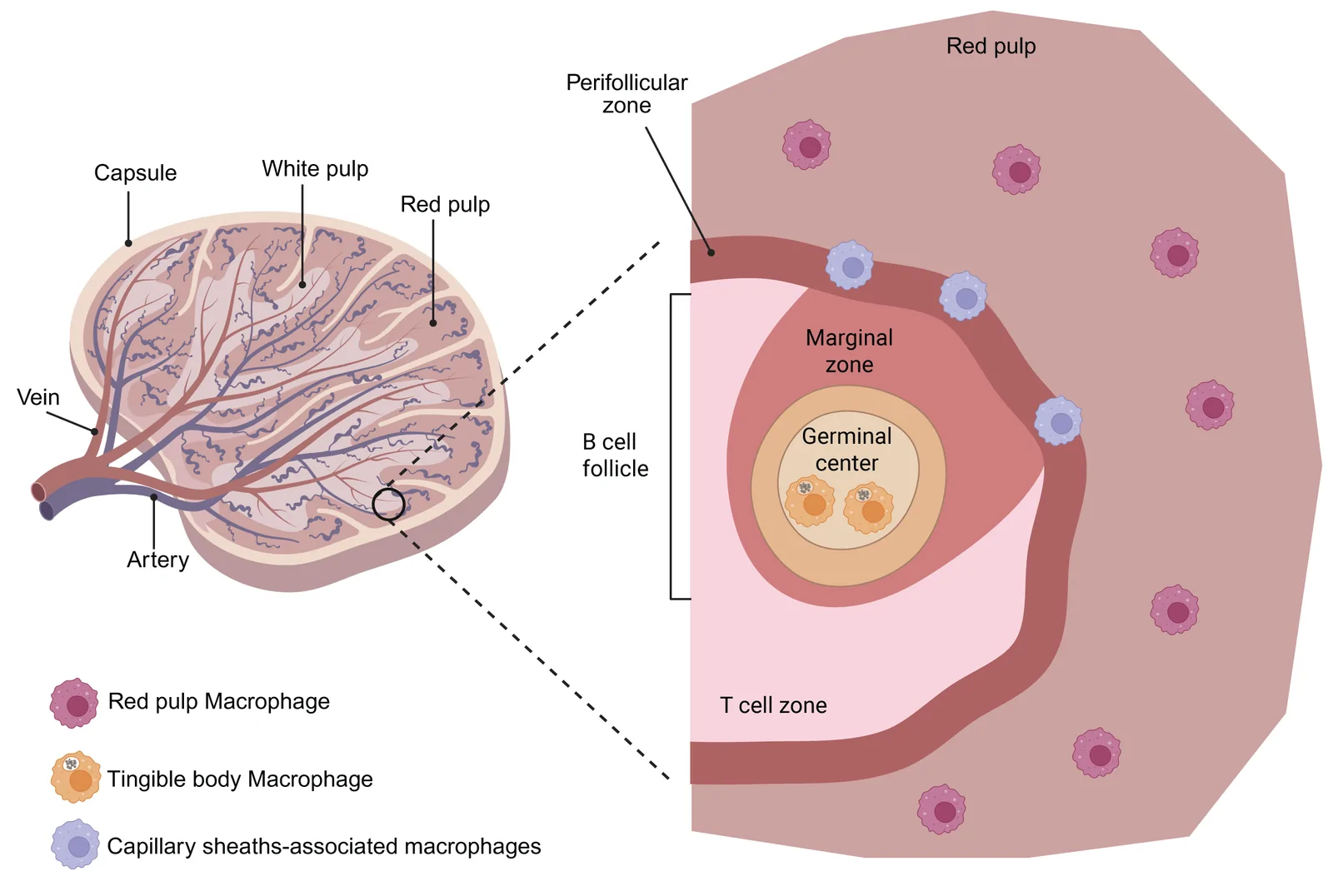
Human spleen macrophages Human spleen can be schematically divided into the red pulp and the white pulp. The red pulp displays an open blood circulation. It also includes arterioles and sheathed capillaries (not depicted). The white pulp is made of lymphoid tissue and includes B cell follicles and lymphoid sheaths in the perifollicular zone. Three populations of macrophages have been described based on their anatomical location: red pulp macrophages in the red pulp, capillary sheath-associated macrophages in the perifollicular zone and tingible body macrophages in germinal centers.

Human spleens can be schematically divided into the red pulp and the white pulp. The red pulp displays an open blood circulation and is rich in erythrocytes. It also includes arterioles and sheathed capillaries, which are surrounded by lymphocytes. The white pulp is made of lymphoid tissue and includes peri-arteriolar lymphoid sheaths and follicles. Of note, rodent spleens harbor an additional zone between the red and white pulps, termed ‘marginal zone’ [63]. Distinct macrophage populations have been described in each of these anatomical regions (Figure 1).

Red pulp macrophages represent the most abundant splenic macrophage population in humans. Red pulp macrophages are CD68+ CD11b- CD163+ CD169– [64]. They are specialized for the clearance of aged erythrocytes and the recycling of iron from hemoglobin [65]. In particular, red pulp macrophages highly express the iron-recycling-related molecules HMOX1 and SLC40A1/Ferroportin [65]. Mouse work has shown that red pulp macrophages are imprinted by heme, which induces the expression of the master transcription factor Spi-C [66,67], and are supported by CSF1 produced specifically by red pulp fibroblasts expressing WT1 [68]. This likely also applies to humans, as human red pulp macrophages preferentially express SPIC [28,65] and scRNA-seq analysis of human spleens identified WT1+ red pulp fibroblasts [68].

Capillary sheath-associated macrophages are found in the perifollicular zone close to follicles [69,70]. These macrophages are CD68+ CD163– CD169+ [69,70]. Because these macrophages are positioned where terminal blood enters sheath regions [71], and by analogy with the mouse marginal zone macrophages [72], this population is likely to function as a scavenger of blood-borne antigens, particles, or pathogens. Nevertheless, a direct demonstration for this function is still lacking.

Another population of macrophages has been reported in the white pulp, in particular in follicular germinal centers [73]. These macrophages were termed ‘tingible body macrophages’ because microscopy images showed phagocytosed material inside these macrophages, and they are thought to be specialized for the clearance of apoptotic B cells by analogy with their mouse counterparts. These macrophages remain poorly characterized in humans.

## Lymph node macrophages

Lymph nodes can be schematically divided into three main zones: subcapsular sinus, cortex, and medulla. The subcapsular sinus corresponds to the space between the lymph node capsule and the cortex and allows the transport of lymphatic fluid. The cortex is a lymphocyte-rich zone, with the outer cortex consisting of B cell follicles and the paracortex consisting of a T cell zone. The inner zone is the medulla and comprises the medullary sinuses that collect the lymph, and between sinuses the medullary cord that is rich in lymphocytes including antibody-secreting plasma cells.

**Figure 2:**
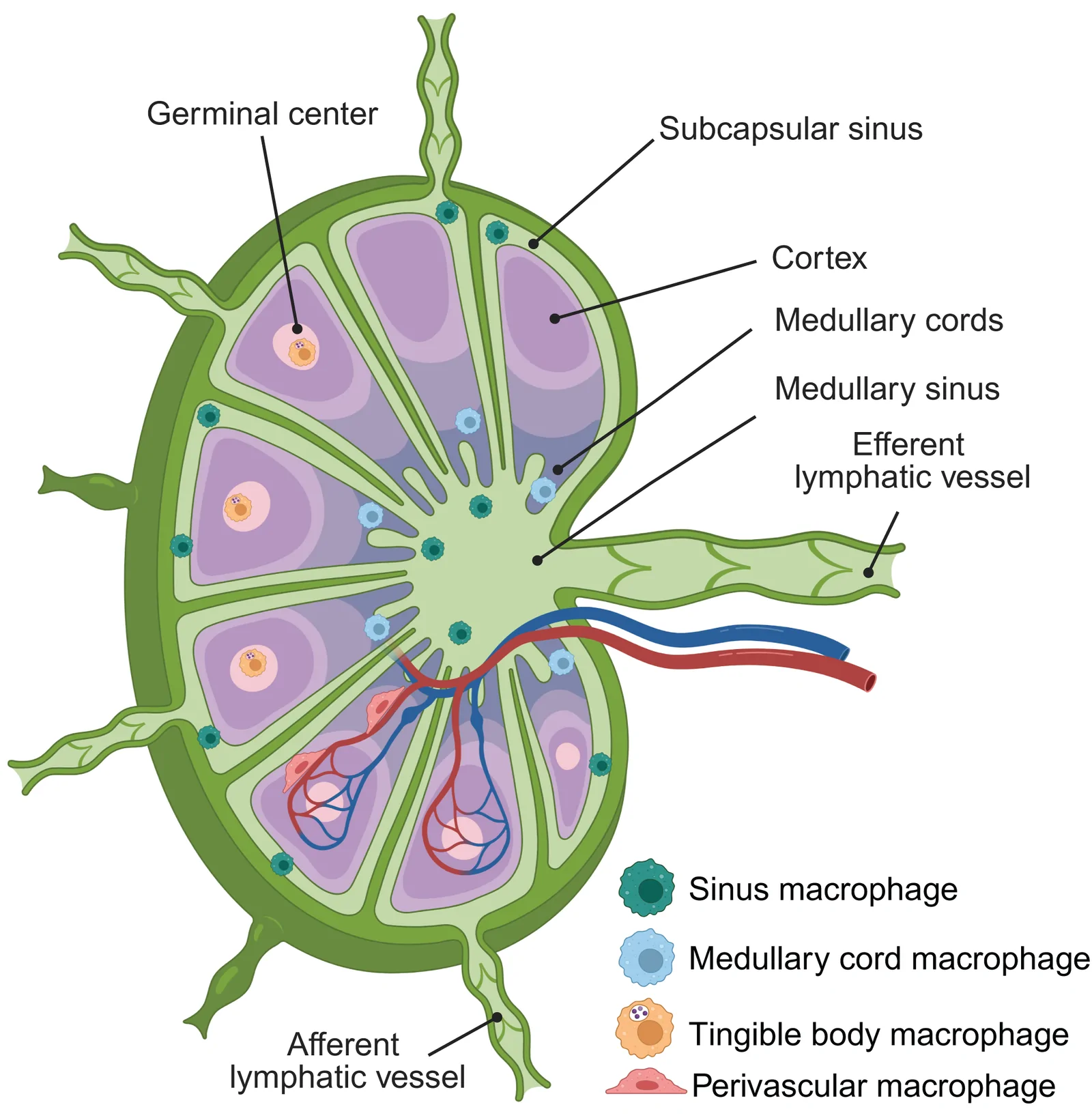
Human lymph node macrophages Human lymph nodes can be schematically divided into three main zones: subcapsular sinus, cortex, and medulla. The subcapsular sinus corresponds to the space between the lymph node capsule and the cortex and allows the transport of lymphatic fluid. The cortex is a lymphocyte-rich zone, with the outer cortex consisting of B cell follicles and the paracortex of a T cell zone. The medulla corresponds to the inner zone and comprises the medullary sinuses that collect the lymph, and the medullary cord in-between sinuses. Four populations of macrophages have been described based on their anatomical location: sinus macrophages, medullary cord macrophages, tingible body macrophages in germinal centers, and perivascular macrophages around blood vessels.

Distinct macrophage populations have been reported in these different zones in human lymph node explants (Figure 2 and Table 1). Studies based on microscopy have identified two major populations: medullary cord macrophages and sinus macrophages. Both populations express pan-macrophage markers including CD68 and CD209/DC-SIGN [74–76]. Medullary cord macrophages are CD163+ CD169– and were reported to be associated with the T cell zone [77]. Sinus macrophages can be subdivided based on their location into subcapsular sinus and medullary sinus macrophages, and appear similar in their phenotype (CD163+ CD169+) [76,78]. More recently, high-dimensional spatial profiling and scRNA-seq analyses have identified additional populations, namely perivascular LYVE1+ macrophages and germinal center macrophages (most likely corresponding to tingible body macrophages) [28,79]. These studies further showed population-specific transcriptomic signatures, confirming that these macrophages represent distinct subsets [28,79].

**Table 1:** Phenotypic markers of human lymph node macrophages

Marker	Perivascular macrophages	Sinus macrophages	Medullary macrophages	Germinal center macrophages
CD68	+	+	+	+
CD163	+	+	+	?
CD169	?	+	−	−
CD209/DC-Sign	?	+	+	+
LYVE-1	+	−	−	−

Reported markers. ? indicates unknown.

In mice, subcapsular sinus macrophages were shown to be specialized for the uptake of lymph-borne antigens and their presentation to B cells [80] or to iNKT cells [81]. The specific functions of human lymph node macrophages remain understudied, but in contrast with the mouse, all populations seem to share the ability to efficiently capture lymph-borne material. Analysis of lung-associated lymph nodes from organ donors of varying ages has found that air-borne particulate matter accumulates with age in lymph node macrophages, presumably after being passively transported through the lymph [77]. More specifically, the medullary cord macrophages were found to contain particles, suggesting for this macrophage population a predominant scavenging function for air-borne particles. The scavenging role of lymph node macrophages is further demonstrated by their ability to capture ink particles from skin tattoos [82]. Using X-ray fluorescence techniques, tattoo pigments were shown to be transported in human skin lymphatics [83]. In skin-draining lymph nodes, tattoo ink was found to accumulate in medullary cord macrophages in both mice and humans, as well as in germinal center macrophages in human [82]. In mice, this was associated with increased macrophage death, inflammation, and altered responses in a vaccination model [82], indicating deleterious effects with broad implications for immune surveillance in tattooed individuals.

CD169+ sinus macrophages have also been proposed to be involved in the uptake of vaccine adjuvants based on a human lymph node explant model [84]. Consistent with this immune surveillance role, in mouse tumor models, CD169+ sinus macrophages in the tumor-draining lymph nodes were shown to be essential for capturing tumor-derived antigens and promoting anti-tumoral immune responses [85–87]. This property may be conserved in humans as clinical studies have shown that a high density of CD169+ macrophages in tumor-draining lymph nodes is associated with a good prognosis in several types of solid tumors [88–90]. However, this property may be context-dependent, as a recent study showed an immunosuppressive role for medullary sinus macrophages in tumor models in mice [91]. Finally, direct evidence that lymph-borne antigens can be presented by human lymph node macrophages to either adaptive or innate lymphocytes is still lacking.

Of note, macrophage populations are modified by lymphoma development, with increased abundance of macrophages with a monocyte-related gene signature being reported in high-dimensional analysis of lymph nodes from diffuse large B-cell lymphoma and follicular lymphoma patients [92].

## Tonsil macrophages

Tonsils are the main organs of nasal-associated lymphoid tissue. They comprise a surface crypt epithelium and a cortex composed of B cell follicles with germinal centers and T cell-rich zones between follicles.

**Figure 3:**
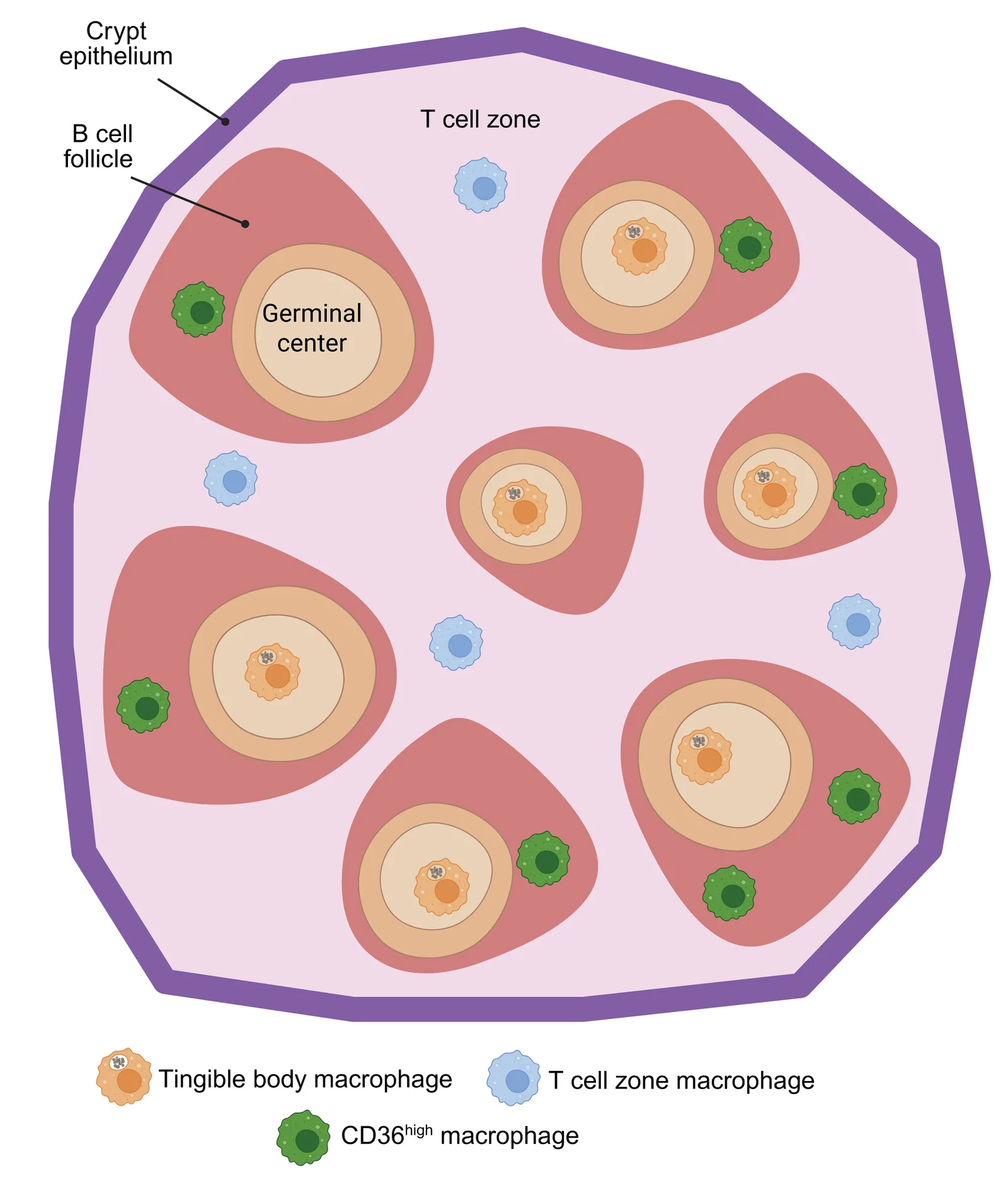
Human tonsil macrophages Human tonsils comprise a surface crypt epithelium and a cortex composed of B cell follicles with germinal centers and T cell-rich zones between follicles. Three populations of macrophages have been described: CD36^high^ macrophages localized in B cell follicles, T cell zone-associated macrophages, and tingible body macrophages in germinal centers.

In non-diseased tonsils, scRNA-seq combined with high-dimensional flow cytometry and imaging has revealed three populations of macrophages displaying distinct transcriptome, phenotype, life cycle, and function [16] (Figure 3). FOLR2+C1QC+CD36^low^ macrophages were found in the T cell zone, while S100A9+CD36^high^ and CD36^int^ macrophages were present in B cell follicles. CD36^high^ macrophages are thought to correspond to short-lived monocyte-derived macrophages due to their lack of proliferative capacity and transcriptomic similarity to monocytes. CD36^int^ macrophages were proposed to represent tingible body macrophages based on their predominant localization in the dark zone of the germinal centers and higher expression of apoptotic cell receptors including MerTK and integrin-β5 [16]. Of note, tingible body macrophages were also identified in tonsil germinal centers in another study using spatial transcriptomics [93].

In terms of functional features, tonsil macrophages (isolated as a bulk population) were characterized for their antigen presentation capacity. While dendritic cells efficiently present exogenous antigens to CD8 T cells on MHC class I molecules (a process termed cross-presentation), macrophages were unable to cross-present antigens [94]. This was linked to the fact that macrophages highly acidify their endocytic compartments, favoring exogenous material degradation, while dendritic cells display less acidic compartments, thereby preserving antigens for subsequent processing [94]. However, in an allogenic setting, tonsil macrophages were shown to stimulate CD4 T cells, although less efficiently than classical dendritic cells, indicating that they can present antigens on MHC class II molecules [95]. More details on the antigen presentation properties of human dendritic cell populations can be found elsewhere [96,97]. In addition, tonsil macrophages, but not macrophages from non-lymphoid tissues, were found to efficiently orient the differentiation of naïve CD4 T cells toward the T follicular helper phenotype, a specialized T cell population required for humoral responses [95]. This property was dependent on the production of the cytokine Activin A by macrophages [95]. Of note, Activin A is essential for the differentiation of human Tfh cells but dispensable for mouse Tfh cells [98], making it impossible to evaluate a similar role for mouse lymphoid organ macrophages in supporting humoral responses. Finally, the CD36^high^ macrophage population was shown to be specialized for Activin A secretion and Tfh stimulation due to the activity of the transcription factor IRF1 [16].

Longitudinal analysis of tonsil biopsies of SARS-Cov-2-infected patients showed that monocytes were increased in tonsils during active infection and display an inflammatory transcriptomic profile [39]. In the present study, two populations of macrophages were identified (*FOLR2*+ and *C1Q*^high^), localizing predominantly in the T cell zone. Tingible body macrophages were hardly detected, possibly due to technical limitations. The proportion of *FOLR2*+ macrophages was increased in tonsils of convalescent patients, with a transcriptomic profile enriched for anti-inflammatory genes, including *IL10*. *FOLR2*+ macrophages were therefore proposed to be involved in the resolution of inflammation in tonsils following acute infection, but this would have to be validated at the functional level.

## Conclusion and future directions

Technological developments in the past few years, in particular single-cell transcriptomics, have enabled major advances in our understanding of macrophage heterogeneity in human tissues, including lymphoid organs. A caveat of these approaches is the need for tissue dissociation, which can generate artifacts. In particular, enzymatic digestion may recover only a fraction of macrophages and can induce macrophage activation during isolation, generating artificial heterogeneity in the population analyzed [99]. Spatial omics approaches are essential to complement these findings and to validate macrophage niches across anatomical sites. In addition, spatial mapping should help resolve whether an equivalent population to mouse marginal zone macrophages exists in humans.

Schematically, the functions of human lymphoid organ macrophages can be divided into scavenging/clearing and supporting immune responses (in particular humoral responses). While the scavenging features align with mouse studies, the role of lymphoid organ macrophages in immune responses may not be conserved in mice due to interspecies differences in the molecular mechanisms involved. Therefore, a more refined mechanistic insight from human macrophage functions is needed. Organotypic or explant models should be used to directly test the role of lymphoid organ macrophages in presenting blood- or lymph-borne antigens. This would be essential for evaluating the potential of macrophage-targeting therapeutic strategies.

Moreover, the functional characteristics of human spleen and lymph node macrophages remain poorly understood. Gaining deeper insight into their biology would have major implications for optimizing vaccine efficacy, including the choice of administration route to engage lymphoid organ macrophages. This knowledge would also improve our understanding of macrophage dysfunction in disease, particularly that of red pulp macrophages in conditions such as thalassemia, sickle cell disease, and hemolytic anemia.

Finally, a major gap in our knowledge of human macrophage biology remains the impact of trained immunity on TRM and the longevity of this phenomenon. This is due to the fact that most mechanistic studies rely on circulating monocytes or *in vitro*-generated monocyte-derived macrophages. However, *in vitro* findings may not always apply to tissue-adapted resident macrophages. Sampling human tissue macrophages longitudinally would be needed to address this gap. In addition, whether trained immunity affects the scavenging efficacy and surveillance role of lymphoid organ macrophages remains unknown. Maladaptive trained immunity in red pulp macrophages could explain in particular the susceptibility to infections by encapsulated organisms of patients chronically exposed to heme and free hemoglobin [100]. It could also help identify macrophage-based biomarkers of splenic dysfunction and guide therapeutic decisions around splenectomy. Finally, in the context of cancer, understanding trained immunity of human lymph node macrophages could support strategies to reprogram macrophages toward a more anti-tumoral phenotype [101], potentially synergizing with checkpoint inhibitors or other immunotherapies.

## Open Access

Open access for this article was enabled by the participation of INSERM in an all-inclusive Read & Publish agreement with Portland Press and the Biochemical Society.
